# The Anticancer Effects of the Pro-Apoptotic Benzofuran-Isatin Conjugate (5a) Are Associated With p53 Upregulation and Enhancement of Conventional Chemotherapeutic Drug Efficiency in Colorectal Cancer Cell Lines

**DOI:** 10.3389/fphar.2022.923398

**Published:** 2022-08-15

**Authors:** Mansoor-Ali Vaali-Mohammed, Maha-Hamadien Abdulla, Sabine Matou-Nasri, Wagdy M. Eldehna, M. Meeramaideen, Eslam B. Elkaeed, Mohammed El-Watidy, Noura S. Alhassan, Khayal Alkhaya, Omar Al Obeed

**Affiliations:** ^1^ Colorectal Research Chair, Department of Surgery, King Saud University College of Medicine, Riyadh, Saudi Arabia; ^2^ Department of Zoology, Jamal Mohamed College (Autonomous), Affiliated to Bharathidasan University, Tiruchirappalli, India; ^3^ King Abdullah International Medical Research Center, Cell and Gene Therapy Group, Medical Genomics Research Department, Ministry of National Guard Health Affairs, King Saud bin Abdulaziz University for Health Sciences, Riyadh, Saudi Arabia; ^4^ School of Biotechnology, Badr University in Cairo, Badr, Egypt; ^5^ Department of Pharmaceutical Chemistry, Faculty of Pharmacy, Kafrelsheikh University, Kafrelsheikh, Egypt; ^6^ Department of Pharmaceutical Sciences, College of Pharmacy, AlMaarefa University, Riyadh, Saudi Arabia; ^7^ College of Medicine Research Center (CMRC), King Saud University College of Medicine, Riyadh, Saudi Arabia

**Keywords:** colorectal cancer, epithelial-mesenchymal transition, synthetic compound, apoptosis, cell cycle, anticancer

## Abstract

The present study aimed to investigate in-depth a cytotoxic novel benzofuran-isatin conjugate (5a, 3-methyl-N'-(2-oxoindolin-3-ylidene)benzofuran-2-carbohydrazide) with promising potential anticancer activities in colorectal adenocarcinoma HT29 and metastatic colorectal cancer (CRC) SW620 cell lines. Thus, the primary cell events involved in tumorigenicity, tumor development, metastasis, and chemotherapy response were explored. Both CRC cell lines were exposed to different concentrations of Compound 5a and then subjected to real-time cell viability, migration, and invasion assays, colony formation and cytotoxicity assays, and flow cytometry for cell cycle analysis and apoptosis determination. Western blot and RT-qPCR were performed to assess the protein and transcript expression levels of epithelial-mesenchymal transition (EMT), cell cycle, and apoptosis markers. We showed that the Compound 5a treatment exhibited anticancer effects through inhibition of HT29 and SW620 cell viability, migration, and invasion, in a dose-dependent manner, which were associated with the upregulation of the tumor suppressor p53. Compound 5a also inhibited the colony formation ability of HT29 and SW620 cells and reversed EMT markers E-cadherin and N-cadherin expression. CRC cell exposure to Compound 5a resulted in a cell cycle arrest at the G1/G0 phase in HT29 cells and at the G2/M phase in SW620 cells, along with the downregulation of cyclin A1 expression, described to be involved in the S phase entry. Furthermore, Compound 5a-induced apoptosis was associated with the downregulation of the anti-apoptotic Bcl-xl marker, upregulation of pro-apoptotic Bax and cytochrome c markers, and increased mitochondrial outer membrane permeability, suggesting the involvement of mitochondria-dependent apoptosis pathway. In addition, the combination studies of Compound 5a with the main conventional chemotherapeutic drugs 5-fluorouracil, irinotecan, and oxaliplatin showed a more potent cytotoxic effect in both CRC cells than a single treatment. In conclusion, our findings described the interesting *in vitro* anticancer properties of Compound 5a, shown to have possible antitumor, antimetastatic, and pro-apoptotic activities, with the enhancement of the cytotoxic efficiency of conventional chemotherapeutic drugs. *In vivo* studies are requested to confirm the promising anticancer potential of Compound 5a for CRC therapy.

## Introduction

Cancer, the most complex heterogeneous disease occurred due to the failure of controlled cell division and differentiation mechanisms, can invade vascularized organs and aggressively spread misrepresentation of the tumors (Fouad and Aanei, 2017). Colorectal cancer (CRC) is the third leading cause of cancer-related death in most developed countries and accounts for ∼10% of all cancers (Siegel et al., 2021). Hyperproliferative colonic epithelial cancer cells result in benign adenoma, which further evolves into carcinoma, the malignant tumor (Smit et al., 2020). Activated cancer cells developing high invasive potential contribute to tumor spread through metastasis process via blood vessels and lymphatic system (Pretzsch et al., 2019). Metastatic CRC (mCRC) patients, clinically described for the presentation of distant lymph node metastasis, have a high mortality rate (Ishii et al., 2022).

Most of the cytostatic anticancer drugs, including alkylating agents (e.g., oxaliplatin), antimetabolites (e.g., 5-fluorouracil), anthracyclines (e.g., doxorubicin), and topoisomerase inhibitors (e.g., irinotecan) target DNA synthesis and replication machinery (De Almeida et al., 2021). Chemotherapy drug administration, in single or combined treatments, is optimized by finding a balance between killing cancer cells and sparing the normal cells (Liu et al., 2015). Anticancer treatment approaches often accompany severe side effects due to the unspecific cytotoxicity of cancer and destroying normal cells into immunogenic necrotic cells (Redondo-Blanco et al., 2017; Wang et al., 2018). Hence, the search for safe anticancer drugs promoting apoptosis, the natural programmed cell death, and exhibiting antitumorigenic, anti-invasion, and antimetastatic activities has been intensified (Abraha and Ketema, 2016; Anderson et al., 2019). During tumor progression, cancer cells display cellular plasticity driven by molecular and phenotypic changes and subsequently present epithelial-mesenchymal transition, an important feature contributing to metastasis and chemotherapy resistance (Bakir et al., 2020; De Las Rivas et al., 2021). Lately, a new class of antimetastatic and anti-invasion synthetic and natural product-based drugs has emerged, targeting mainly kinases involved in actomyosin cytoskeleton-mediated contractility (Gandalovičová et al., 2017).

Alternatively, novel therapeutic approaches targeting specific checkpoints, essential for the controlled regulation of cancer cell proliferation, cell cycle regulation, and apoptosis markers such as deregulated, mutated, or overexpressed related proteins, and selectively affect cancer cells or their surrounding environment with negligible effects on normal cells, have been developed (Zhong et al., 2021). The increased selective treatment plans towards colon cancer cells have received more consideration and continuing active search. Benzofuran and several of its derivatives have been reported to exhibit cytotoxicity and have therapeutic potential as anticancer agents for colon, lung, and breast cancers (Eldehna et al., 2021a; Al-Sanea et al., 2021; Eldehna et al., 2021b).

Isatin (indole-2,3-dione) is a well-known organic compound derived from indole widespread in human and other mammalian fluids and tissues and found in various natural products such as alkaloids, marines, and fungal metabolites (Varun et al., 2019; Nath et al., 2021). Sunitinib and Nintedanib are examples of isatin-based clinically approved drugs (Rossi et al., 2017). In the current medical era, Isatin is an important privileged scaffold in anticancer drug discovery and development (Hou et al., 2020). Moreover, several recent studies have adopted the hybridization approach to conjugate the isatin moiety with diverse heterocycles to develop promising anticancer molecules (El-Naggar et al., 2018; Eldehna et al., 2021a; Eldehna et al., 2021b). Recently we have reported a series of benzofuran-isatin conjugates as a novel anticancer agent with apoptosis-inducing molecular mechanisms using colon cancer cell lines (Eldehna et al., 2021c). In particular, Compound 5a (3-methyl-N'-(2-oxoindolin-3-ylidene)benzofuran-2-carbohydrazide) displayed potent and selective anti-proliferative action towards SW620 and HT29 CRC cell lines and provoked apoptosis in a dose-dependent manner in SW620 cells (Eldehna et al., 2021c).

In the present study, we aimed to verify whether the Benzofuran derivative referred to as Compound 5a could exert anticancer effects against two human CRC cell lines, the human colorectal adenocarcinoma HT29 and metastatic colorectal cancer (mCRC) SW620 cell lines by inhibiting cell viability, migration, invasion, and colony formation. We also investigated the molecular mechanisms involved in the Compound 5a-induced cell death. We examined the potential potentiation of standard chemotherapeutic drugs (i.e., irinotecan, 5-fluorouracil, oxaliplatin) cytotoxic efficiency following combined treatments with Compound 5a.

## Materials and Methods

### Reagents

Cell culture media and related reagents were provided by Thermo Fischer Scientific (Eugene, OR, United States). Annexin V/Dead Cell Apoptosis kit (cat. no. V13242) and Rhodamine 123 were procured from Molecular Probes (Thermo Fischer Scientific). Fluorouracil (also known as 5-FU), oxaliplatin (also known as L-OHP), and Camptosar^®^ (also known as irinotecan, IRI) were purchased from Hospira Inc. (Lake Forest, IL, United States). 3-(4, 5-dimethyl thiazolyl-2)-2, 5-diphenyltetrazolium bromide (MTT, cat. no. M5655), paraformaldehyde (cat. no. 47608), propidium iodide (PI, cat. no. 4864), protease inhibitors (cat. no. 535140), and crystal violet (cat. no. C0775) were provided by Sigma-Aldrich (St. Louis, MO, United States). RNase A (10 mg/ml) was procured from Qiagen (Hilden, Germany) and Clarity Western ECL Substrate and Bradford protein assay from Bio-Rad Laboratories (Hercules, CA, United States). Primary antibodies directed against E-cadherin (cat. no. sc-71008), N-cadherin (cat. no. sc-59987), anti-cyclin A1 (cat. no. sc-271645), anti-cyclin B1 (cat. no. sc-70898), anti-cyclin D1 (cat. no. sc-753), anti-cytochrome c (cat. no. sc-13156), Bcl-xl (cat. no. sc-8392), Bax (cat. no. sc-70408), p53 (cat. no. sc-47698), and *ß*-Actin (cat. no. sc-47778) were purchased from Santa Cruz Biotechnology, Inc. (Dallas, TX, United States).

### Synthesis of 3-Methyl-N'-(2-Oxoindolin-3-Ylidene)Benzofuran-2-Carbohydrazide (5a)

Intermediates 2 and 3 and Compound 5a were reported previously (Eldehna et al., 2021c). In brief, 3-methyl benzofuran-2-carbohydrazide 3 (0.09 g, 0.5 mmol) was dissolved in absolute ethanol (5 ml) and catalytic drops of glacial acetic acid, then isatin 4a (0.07 g, 0.5 mmol) was added, and then the reaction mixture was refluxed for 5 h. After cooling, the precipitate was filtered, washed with water (3 × 5 ml) then recrystallized from acetic acid to furnish Compound 5a.

Melting point >300°C; IR: 3301, 3250 (2NH) and 1711, 1693 (2C = O); 1H NMR (DMSO-d6, 400 MHz) *δ* ppm: 2.64 (s, 3H, CH3), 6.96 (t, 1H, Ar-H, J = 8.4 Hz), 7.12–7.22 (m, 1H, Ar-H), 7.40–7.49 (m, 2H, Ar-H), 7.57–7.64 (m, 1.7H, Ar-H), 7.69 (d, 0.3H, Ar-H, J = 8.4 Hz), 7.78 (d, 0.7H, Ar-H, J = 8.4 Hz), 7.84 (d, 1H, Ar-H, J = 7.6 Hz), 7.93 (d, 0.3H, Ar-H, J = 7.6 Hz), 10.91, 11.60 (2s, 1H, NH isatin), 11.37, 14.05 (2s, 1H, NH); 13C NMR (DMSO-d6, 101 MHz) *δ* ppm: 9.43, 9.46 (CH3), 111.41, 111.72, 112.32, 112.59, 116.21, 120.31, 121.55, 121.98, 122.07, 122.56, 123.22, 124.13, 124.25, 125.02, 125.80, 126.77, 128.64, 129.00, 129.26, 132.42, 133.59, 138.85, 141.67, 142.16, 142.70, 143.13, 144.53, 153.49, 153.66, 156.30, 163.30, 165.05; MS m/z [%]: 319 [M+, 83]; Analytically calculated for C18H13N3O3 (319.3): C, 67.71; H, 4.10; N, 13.16; found C, 67.77; H, 4.14; N, 13.11.

### Cell Culture

Human normal colon epithelial cell (CCD841 CoTr), colorectal adenocarcinoma HT29, and mCRC SW620 cell lines were obtained from American Type Culture Collection (ATCC, Manassas, VA, United States) and grown in a complete medium composed of DMEM supplemented with 10% heat-inactivated fetal bovine serum (FBS), 100 μg/ml streptomycin, 100 IU/ml penicillin and 2 mmol/l L-glutamine. The cells were cultured at 37°C in a saturated air humidity 5% CO2-incubator. At confluence, the cells were passaged every 2–3 days using enzymatic digestion with 0.1% trypsin/0.02% EDTA and split at a ratio of 1:2 or 1:3.

### Cytotoxicity Assay

Cytotoxicity was evaluated using a 3-(4, 5-dimethyl thiazolyl-2)-2, 5-diphenyltetrazolium bromide (MTT) assay based on the reduction of the tetrazolium dye by mitochondrial NAD(P)H-dependent oxidoreductase enzymes, highly active in the viable cells (Al-Khayal et al., 2017). Briefly, HT29 and SW620 cells were seeded in 96-well plates (Falcon™, Thermo Fisher Scientific). The next day, the cells were treated with various concentrations of Compound 5a (1.25–30 µM) alone, various concentrations (1–20 μM) of chemotherapeutic drugs (i.e., irinotecan, 5-fluorouridine, and oxaliplatin) alone, or with equimolar mixtures of Compound 5a with each chemotherapeutic drug. After 24 h incubation, 10 μL of freshly prepared MTT solution (5 mg/ml in phosphate-buffered saline, PBS) was added to the cells, followed by a further 3-h incubation, which resulted in the formation of formazan crystals. These crystals were then dissolved by adding and mixing 100 μL of dimethyl sulfoxide (DMSO) in each well for 15 min incubation. The absorbance of the reduced tetrazolium dye was measured at 540 nm using the ELx800™ microplate reader (BioTek Instruments, Inc., Winooski, VT, United States). Three independent experiments were performed, and each experiment was done in triplicate for each condition.

### Real-Time Cell Proliferation, Migration and Invasion by the xCELLigence System

The effect of Compound 5a on HT29 and SW620 cell proliferation, migration, and invasion were assessed in real-time using the xCELLigence Real-Time Cell Analyzer Dual Plate (RTCA-DP) system according to the manufacturer’s recommendations (Acea, Biosciences Inc. United States). Briefly, HT29 cells (5 × 103/well) and metastatic SW620 cells (12 × 103/well) in 150 µL medium/well were cultured in a 16-well E-plate (ACEA Biosciences Inc., San Diego, CA, United States) for cell proliferation assay and CIM-plate for cell migration and invasion assays. Both cell lines were treated with (5, 10, 20 μM) and without (i.e., the control) the. Compound 5a then were subjected to serum starvation 1 h before the start of the measurement. For the cell proliferation assay, the cell treatment was performed the next day of the cell seeding. For the cell migration assay, the lower chamber was loaded with DMEM supplemented with 10% FBS. For cell invasion assay, collagen (50 μg/ml)-coated CIM-plates were used. Cell index values were monitored every 15 min for 30 h, every 15 min for 170, and every 10 min for 120 h for cell proliferation, migration, and invasion assays, respectively. For cell proliferation assessment, baseline cell indices were calculated for at least two measurements from three replicate experiments. At least three independent experiments were performed to monitor cell migration and invasion, and each assay was carried out in triplicates.

### Colony Formation Assay

Colony formation assay was done as previously described by Al-Khayal et al. (2020). HT29 and SW620 cells were harvested and re-suspended in complete media. The cells were seeded in 6-well plates at 500 cells/well containing 2.0 ml media and incubated for 4–6 h to allow cell attachment. Different concentrations (5, 10, 20 μM) of Compound 5a were added for 24 h incubation. The next day, the medium containing Compound 5a was replaced with a fresh medium and further incubated for 10–14 days. Colonies were fixed with 4% paraformaldehyde and stained with 0.05% crystal violet. The colonies were quantified using an inverted light Sundew MCX1600 microscope (MICROS, Vienna, Austria).

### Cell Cycle, Apoptosis, and Mitochondrial Membrane Potential Activity Analyses by Flow Cytometry

Both HT29 and SW620 cells (2 × 105/well) were seeded in 6-well plates. The next day, the cells were treated with or without Compound 5a and tested at different concentrations (5, 10, 20 μM) for 24 h incubation. The cells were then subjected to flow cytometry analysis for cell cycle analysis, apoptosis status determination, and mitochondrial membrane potential assessment.

For cell cycle analysis, the untreated and treated cells were harvested, and the attached and floating cells were collected. After centrifugation, the cells were fixed by slowly dropping 70% ice-cold ethanol and left for at least 30 min at 4°C. The cells were washed twice with ice-cold PBS through centrifugation (2000 rpm, 5 min). RNase A (10 mg/ml) and PI (50 μg/ml) were added to the cell suspension for 10 min at room temperature. Cell cycle phases (Sub G, G0/G1, S, and G2/M phases) were analyzed using a Becton Dickinson (BD) FACSCalibur™ cell analyzer (BD Biosciences, Franklin Lakes, NJ, United States) equipped with CellQuest™ software Pro version 6.0 at an emission >575 nm (FL3 channel) and 10,000 events were gated for each test.

Apoptosis and necrosis status were determined as reported previously (Al-Khayal et al., 2017). The detection of apoptosis was performed using the Annexin V/Dead Cell Apoptosis kit as recommended by the manufacturer’s instructions. The untreated and treated cells were re-suspended in Annexin V binding buffer and incubated at 25°C with Annexin V-fluorescein isothiocyanate (FITC; 5 µL) propidium iodide (PI; 1 µL) for 15 min. Data acquisition and analysis were performed using CellQuest™ software. Studies were completed wherein fluorescence emission was measured at 530 nm (FL1 channel) for FITC Annexin V and >575 nm (FL3 Channel) for PI, and 10,000 events were gated for each test.

For MtMP activity assessment, the non-treated and treated cells were harvested, washed twice with PBS, and incubated for 20 min at 37°C with the positively charged membrane-permeant fluorescent dye Rhodamine 123 (Rhod123, 25 ng/ml), used as voltage reporter for mitochondrial membrane potential. Rhod123-positive cells were detected using flow cytometry as previously described (Al-Khayal et al., 2017).

### RNA Extraction and Quantitative Real-Time Reverse Transcription-Polymerase Chain Reaction

Both HT29 and SW620 cells (1 × 106/dish) were cultured in a 100-mm dish up to 60% confluency in the complete medium for 24 h incubation. The next day, the cells were treated with 10 μM of Compound 5a for further 24-h incubation. Total RNA was extracted from treated and untreated cells using a PARIS™ kit (Ambion Inc., Austin, TX, United States). A High-Capacity cDNA kit (cat. no. 4368814) was used for reverse transcription (Applied Biosystems, Waltham, MA, United States). RNA quality was evaluated by assessing the A260/280 ratio (1.8–2.0) using a NanoDrop ND-2000 UV-VIS spectrophotometer (Thermo Fisher Scientific, Inc.).

Quantitative PCR analysis was performed on ViiA™ 7 real-time PCR system (Thermo Fisher Scientific) using the SYBR Green PCR Master Mix (cat no 4385612, Thermo Fisher Scientific). The relative mRNA expression levels of E-cadherin, N-cadherin, CyclinA1, Cytochrome c, Bcl-xl, Bax, and p53 were normalized to GAPDH for quantitative real-time reverse transcription-polymerase chain reaction (qRT-PCR). The primers sequences are listed in Table 1. For each gene analysis, a negative control was prepared without a cDNA template. All the reactions were performed in triplicate and repeated in each experiment three times separately.

**TABLE 1:** Primer sequences.

Target Genes	Primer Sequences
E-cadherin	F: 5′ -ACC​AGA​ATA​AAG​ACC​AAG​TGA​CCA-3′
	R: 5′- AGC​AAG​AGC​AGC​AGA​ATC​AGA​AT -3′
N-cadherin	F: 5′-ATT​GGA​CCA​TCA​CTC​GGC​TTA-3′
	R: 5′-CAC​ACT​GGC​AAA​CCT​TCA​CG-3′
Cyclin A1	F: 5′-G TCA​CCA​CAT​ACT​ATG​GAC​ATG-3′
	R: 5′-AAG​TTT​TCC​TCT​CAG​CAC​TGA​C-3′
Cyclin B1	F: 5′-AAA​GGC​GTA​ACT​CGA​ATG​GA-3′
	R: 5′-CCG​ACC​TTT​TAT​TGA​AGA​GCA-3′
Cyclin D1	F: 5′-TCC​AGA​GTG​ATC​AAG​TGT​GA-3′
	R: 5′-GAT​GTC​CAC​GTC​CCG​CAC​GT-3′
Cytochrome c	F: 5′-GGC​TGC​AGT​GTA​GCT​GTG​AT-3′
	R: 5′-GAT​GGA​GTT​TCC​TTT​ATC​TGT​TGC-3′
PARP	F: 5′-GGA​AAG​GGA​TCT​ACT​TTG​CCG-3′
	R: 5′-TCG​GGT​CTC​CCT​GAG​ATG​TG-3′
Bcl-2	F: 5′-AAG​ATT​GAT​GGG​ATC​GTT​GC-3′
	R: 5′-GCG​GAA​CAC​TTG​ATT​CTG​GT-3′
Bcl-xl	F: 5′-CCC​AGA​AAG​GAT​ACA​GCT​GG-3′
	R: 5′-GCG​ATC​CGA​CTC​ACC​AAT​AC-3′
Bax	F: 5′-GTG​CAC​CAA​GGT​GCC​GGA​AC-3′
	R: 5′-TCA​GCC​CAT​CTT​CTT​CCA​GA-3′
p53	F: 5′-GAG​GTT​GGC​TCT​GAC​TGT​ACC-3′
	R: 5′-TCC​GTC​CCA​GTA​GAT​TAC​CAC-3′
GAPDH	F: 5′-AAGGTCGG AGTCAACGGATTTGGT-3′
	R: 5′-ATG​GCA​TGG​ACT​GTG​GTC​ATA​GT-3′

### Cell Lysate Preparation and Immunoblotting Analysis

Both HT29 and SW620 (1 × 106/well) cells were seeded up to 60% confluency and cultured in the complete medium for 24 h. The cells were treated with (5–10 μM) or without Compound 5a for 24 h incubation. The cells were then washed with 1 × PBS, harvested, and lysed in ice-cold RIPA lysis buffer, combined with protease inhibitors (Al-Obeed et al., 2020). From each whole cell lysate, the total protein concentration was evaluated by the colorimetric Bradford protein assay at 595 nm absorbance using a SmartSpecTM Plus spectrophotometer (Bio-Rad Laboratories). Denatured proteins were loaded on precast TGX gels. They were analyzed by immunoblotting with primary antibodies directed against E-cadherin, N-cadherin, cyclin A1, cyclin B1, cyclin D1, cytochrome c, Bcl-xl, Bax, p53, and *ß*-Actin. Reactivity was detected with horseradish peroxidase-conjugated secondary antibodies and visualized through chemiluminescence by Clarity Western ECL Substrate (Bio-Rad Laboratories). The probed PVDF membranes were developed using C-DiGit Blot Scanner (LI-COR, Hamburg, Germany).

### Statistical Analysis

The results are presented as mean ± standard deviation (SD) of three independent experiments. The student’s t-test was used for statistical analysis, and a one-way ANOVA statistical test was applied to study the difference between the control and treated groups. The *p*-values lower than 0.05 were considered statistically significant.

## Results

### Synthesis of Compound 5a

**FIGURE 1:**
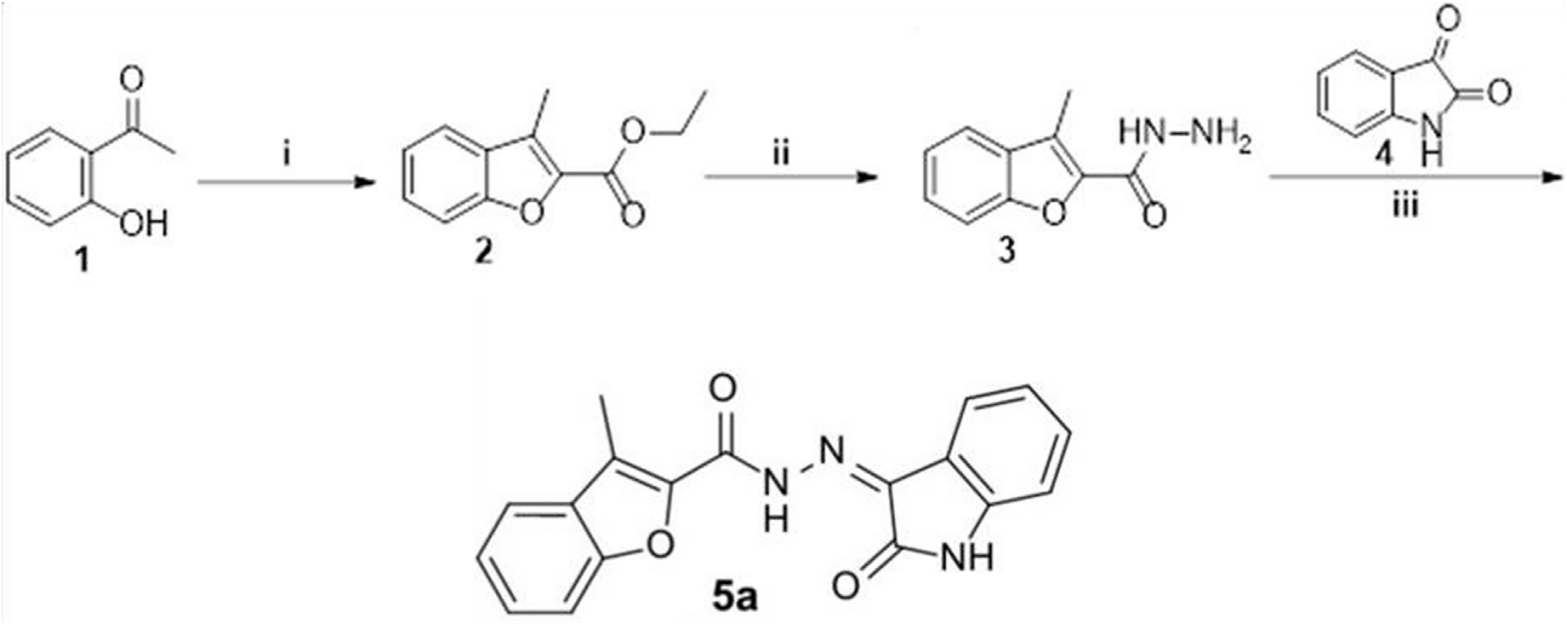
Synthesis of Compound 5a. (i) Ethyl 2-bromoacetate, anhydrous acetonitrile, K2CO3, reflux 8 h; (ii) hydrazine hydrate, methyl alcohol, reflux 4 h; (iii) ethanol/AcOH (Cat.)/reflux 5 h.

The synthetic strategy to prepare Compound 5a is depicted in Figure 1. First, 2′-hydroxy acetophenone 1 was cyclized *via* the reaction with ethyl bromoacetate in refluxing acetonitrile in the presence of potassium carbonate to furnish the ester derivative 3-methylbenzofuran-2-carboxylate 2. Then, ester 2 was heated with hydrazine hydrate in refluxing methyl alcohol to produce the hydrazide intermediate 3-methyl benzofuran-2-carbohydrazide 3, which subsequently condensed with isatin 4 in refluxing the ethanol and in the presence of catalytic drops of glacial acetic acid to afford Compound 5a in 80% yield.

### Compound 5a Reduced the Real-Time CRC Cell Proliferation, Migration, Invasion, and Upregulated Tumor Suppressor p53

**FIGURE 2:**
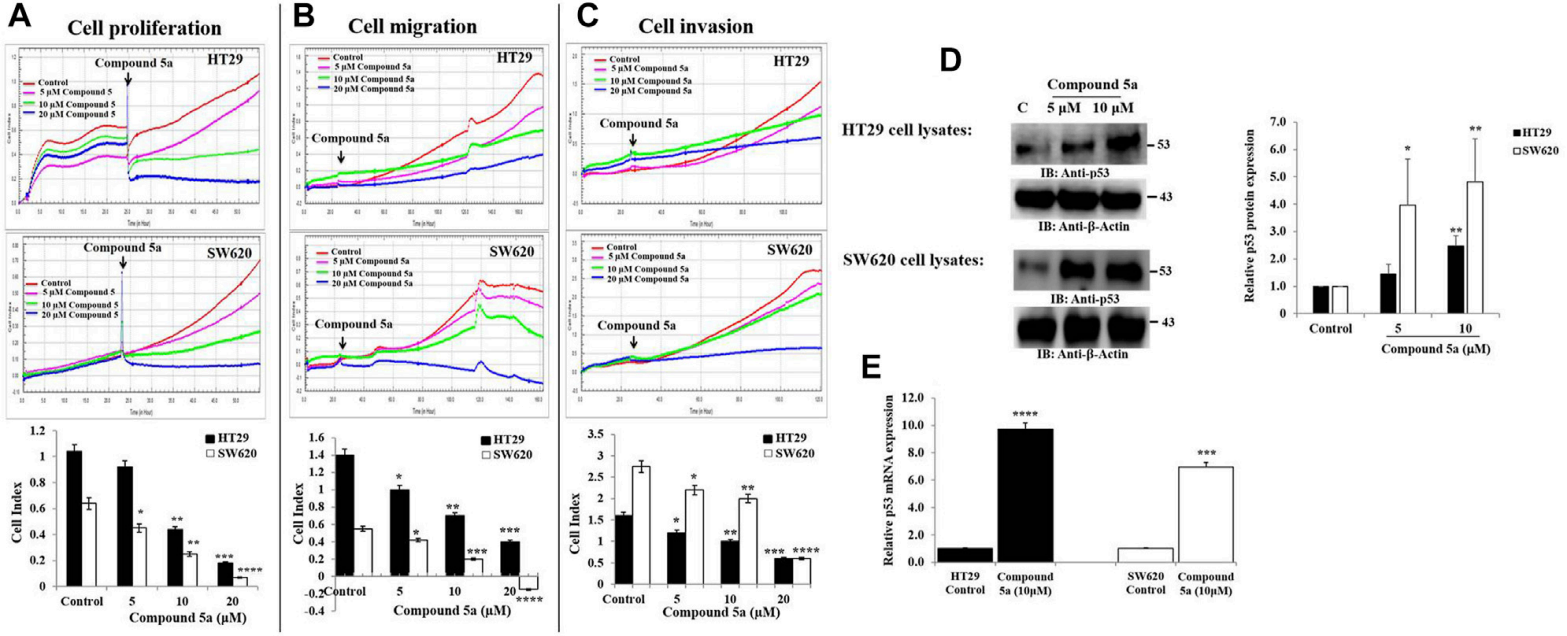
Compound 5a inhibits adenocarcinoma HT29 and metastatic CRC SW620 cell proliferation, migration, and invasion and upregulates p53 protein and gene expression in both cell lines. **(A)** Real-time cell proliferation was monitored by measuring cell indexes in control and treated cells to assess the anti-proliferative effect of Compound 5a using the xCELLigence RTCA-DP system. **(B)** Real-time cell migration and cell invasion **(C)** were monitored using the xCELLigence RTCA-DP system. Bar graphs showing the results expressed as mean ± SD of three independent experiments (*n* = 3). **(D)** Total HT29 and SW620 cell lysates were immunoblotted against the protein p53 using a monoclonal anti-p53 antibody. Densitometry analysis was conducted as follows: Intensity of the protein bands was semi-quantified, normalized to the control, and plotted as relative protein expression to the loading control *ß*-actin. The bar graph indicates the relative p53 protein expression level as the mean ± SD of three independent experiments. **(E)** Bar graph showing the relative mRNA expression level of p53 related to the internal control GAPDH. Data are shown as mean ± SD (*n* = 3). The data were considered significant when reporting *p < 0.05, **p < 0.01, ***p < 0.001, and ****p < 0.0001 *vs*. Control.

Among 16 benzofuran derivatives synthesized, we reported the most potent cytotoxic effect of Compound 5a on the colorectal adenocarcinoma HT29 and metastatic CRC SW620 cell lines (Eldehna et al., 2021c). In the present study, the Compound 5a-treated CRC cells were subjected to cell-based assays exploring the main cell events involved in cancer development and progression to evaluate the anticancer potential of Compound 5a. Hence, an automated xCELLigence Real-Time Cell Analyzer Dual Plate (RTCA-DP) system was used to monitor the CRC cell proliferation, migration, and invasion in real-time in response to increasing concentrations of Compound 5a. The real-time monitoring system operated based on the electrical impedance measurement, resulting in the record of the cell index (CI) in HT29 and SW620 cells compared with the untreated cells. The CI represents a dimensionless parameter derived as a relative change in measured electrical impedance. The changes in electrical impedances reflect physiological conditions of the cell morphology, adhesion, proliferation, migration, and invasion before and after the cell treatment. Compared with the untreated cells, the addition of Compound 5a significantly reduced the cell proliferation (Figure 2A), migration (Figure 2B), and invasion (Figure 2C) in both CRC cells in a dose-dependent manner (Figure 2). The inhibitory effects of Compound 5a were revealed to be higher regarding SW620 cell proliferation and migration than those exerted on the HT29 cells. However, regarding the cell invasion, the Compound 5a inhibitory effect was higher in HT29 cells than in the metastatic SW620 cells (Figure 2C). Well known as the tumor suppressor, p53 at the protein and transcript levels was assessed using Western blot and RT-qPCR technologies. Upon Compound 5a treatment, p53 protein expression levels significantly increased in HT29 cells when tested at 10 μM (2.46-fold, *p* = 0.0027) and in SW620 cells when tested at 5 μM (2.95-fold, *p* = 0.038) and 10 μM (4.81-fold, *p* = 0.0013), compared with the basal expression level detected in untreated cells (Figure 2D). This upregulation of p53 induced by Compound 5a was confirmed at the gene expression level (Figure 2E).

### Compound 5a Inhibited Colony Formation and Reversed E-Cadherin and N-Cadherin EMT Phenotype Markers in CRC Cells

**FIGURE 3:**
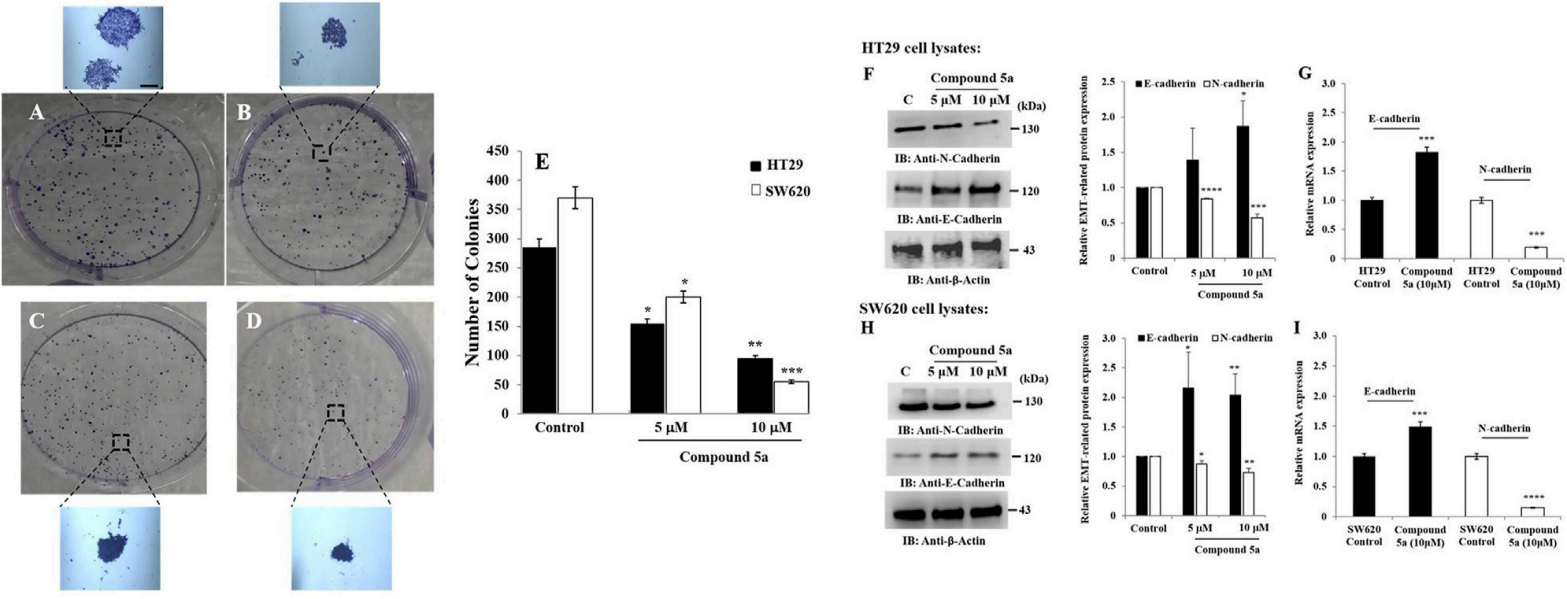
Compound 5a inhibits colony formation of HT29 and SW620 cells and upregulates E-cadherin and N-cadherin expression in both cell lines. Both HT29 **(B)** and SW620 **(D)** cells were further incubated for 10–12 days for colony formation at 37°C, along with untreated HT29 **(A)** and SW620 **(C)** cells. Crystal violet staining was done, and the number of colonies was determined using a light microscope. **(E)** The bar graph depicts the number of colonies, and the data are shown as mean ± SD (*n* = 3). Total HT29 **(F)** and SW620 **(H)** cell lysates were immunoblotted against the indicated antibodies (i.e., N-cadherin and E-cadherin). Bar graph indicating the relative protein expression level related to the loading control *ß*-actin. Bar graph showing the relative mRNA expression level of E-cadherin and N-cadherin related to the internal control GAPDH monitored in HT29 **(G)** and SW620 **(I)** RNA extracts. Data are shown as mean ± SD (*n* = 3). *p < 0.05, **p < 0.01, ***p < 0.001, and ****p < 0.0001 *vs*. Control.

We evaluated the *in vitro* tumorigenic effect of Compound 5a tested at different concentrations (5, 10, 20 µM) on HT29 and SW620 cells using colony formation assay. Based on the number of colonies formed, Compound 5a significantly inhibited HT29 (Figure 3B) and SW620 (Figure 3D) cell-based colony formation by 50% (*p* = 0.039) when tested at 5 μM and by 70% (*p* = 0.009) and 85% (*p* = 0.00093) when tested at 10 μM, respectively, compared with the untreated control cells (Figure 3).

The colony formation occurs through the cancer cell plasticity regulated by EMT markers such as E-cadherin and N-cadherin (Bakir et al., 2020). Thus, we assessed the protein and gene expression levels of E-cadherin and N-cadherin after protein and RNA extraction from untreated and Compound 5a-treated cells using Western blot and RT-qPCR technologies. Compound 5a significantly increased E-cadherin protein expression in HT29 cells when tested at 10 μM (1.87-fold, *p* = 0.014, Figure 3F) and in SW620 cells when tested at 5 μM (2.15-fold, *p* = 0.030, Figure 3H) and 10 μM (2.03-fold, *p* = 0.007, Figure 3H), compared with the basal level of E-cadherin expression detected in untreated cells (Figure 3). In contrast to E-cadherin upregulation, a significant gradual decrease of N-cadherin protein expression was observed in all treated cells exposed to increased Compound 5a concentrations. At the highest concentration tested, at 10 μM, Compound 5a was suppressed by 45% (*p* = 0.0002, Figure 3F) and 30% (*p* = 0.0023, Figure 3H) N-cadherin protein expression levels in HT29 and SW620 cells, respectively, compared with the basal level detected in untreated cells (Figure 3). Upregulation of E-cadherin (1.82-fold, *p* = 0.00092 for HT29, Figure 3G; 1.5-fold, *p* = 0.00091 for SW620, Figure 3I) and downregulation of N-cadherin (0.19-fold, *p* = 0.000124 for HT29, Figure 3G; 0.15-fold, *p* = 0.000011 for SW620, Figure 3I) expressions in both CRC cells exposed to Compound 5a (10 μM) were confirmed at the gene expression level using RT-qPCR, compared with the basal gene expression level monitored in the untreated cells (Figure 3).

### Compound 5a Induced Cell Cycle Arrest at the G0/G1 Phase in HT29 Cells and the G2/M Phase in SW620 Cells, Reduced the Entry Into S Phase and Downregulated Cyclins D1, B1, and A1 Expression

**FIGURE 4:**
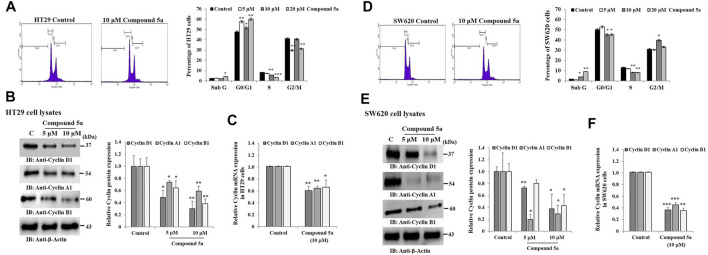
Compounds 5a affects HT29 and SW620 cell cycle progression and downregulates Cyclins D1, A1, and B1. Representative histograms showing the cell cycle profile in untreated (Control) and 10 μM Compound 5a-treated cells. Cell cycle profiles were measured using flow cytometry after PI staining. The bar graph depicts the percentage of HT29 **(A)** and SW620 **(D)** cells distributed in sub G1, G0/G1, S, and G2/M-phases after cell exposure to various concentrations (5-10-20 μM) of Compound 5a. Total HT29 **(B)** and SW620 **(E)** cell lysates were immunoblotted against Cyclins D1, A1, and B1 using a specific monoclonal antibody. Bar graph indicating the relative Cyclins protein expression level, related to the loading control *ß*-actin. Bar graph showing the relative mRNA expression level of Cyclins in HT29 **(C)** and SW620 **(F)** RNA extracts related to the internal control GAPDH. Data are shown as mean ± SD (*n* = 3). *p < 0.05, **p < 0.01, and ***p < 0.001 *vs*. Control.

Cell cycle distribution in (Sub G, G0/G1, S, G2/M) phase was measured by flow cytometry-based on the content of DNA, using propidium iodide (PI) as a DNA-binding dye. The addition of Compound 5a significantly increased the Sub G phase HT29 cell population when tested at 20 µM (64%, *p* = 0.036) and induced cell cycle arrest in G0/G1 phase (∼25%, *p* < 0.01 at 5 and 20 μM), and reduced the cell population in S phase (−33%, *p* = 0.0091 at 10 μM and −75%, *p* = 0.00085 at 20 μM) and G2 phase (−30%, *p* < 0.01 at 5 and 20 μM), compared to the cell cycle distribution monitored in untreated HT29 cells (Figure 4A). Similar results were obtained in SW620 cells exposed to 10 μM of Compound 5a, except that the number of the cells was reduced in G0/G1, and the cell cycle arrest was observed in the G2/M phase (Figure 4D). To address the mechanism responsible for Compound 5a-reduced cell population in the S phase, we assessed the protein expression levels of Cyclin A1 in both HT29 and SW620 cells exposed to 5 and 10 μM of Compound 5a. A significant decrease of Cyclin A1 was observed in HT29 cells (−20%, *p* = 0.012 at 5 μM; −40%, *p* = 0.0091 at 10 μM, Figure 4B) and was more drastic in SW620 cells (−80%, *p* = 0.000016 at 5 μM; −60%, *p* = 0.037 at 10 μM, Figure 4E), compared with the basal expression level of Cyclin A1 detected in untreated cells. Of note, Compound 5a (10 μM) downregulated Cyclin A1 gene expression in both HT29 (−40%, *p* = 0.00935, Figure 4C) and SW620 (−60%, *p* = 0.00081, Figure 4F) cells to a similar extent as detected at the protein expression levels. To address the mechanism responsible for Compound 5a-increased cell population in G0/G1 phase and G2/M phase, we evaluated the protein expression levels of Cyclin D1 and Cyclin B1 in both HT29 and SW620 cells exposed to 5 and 10 μM of Compound 5a. In a dose-dependent manner, Compound 5a significantly decreased Cyclin D1 and Cyclin B1 protein expression levels in both HT29 (Figure 4B) and SW620 (Figure 4E) cells. This downregulation of Cyclin D1 and B1 expression in 10 μM of Compound 5a-treated HT29 (Figure 4C) and SW620 (Figure 4F) cells was also confirmed at the gene expression level.

### Compound 5a Prompted Apoptosis, Downregulated Bcl-Xl, Upregulated Bax and Cytochrome c, and Decreased Mitochondrial Membrane Potential in CRC Cells

**FIGURE 5:**
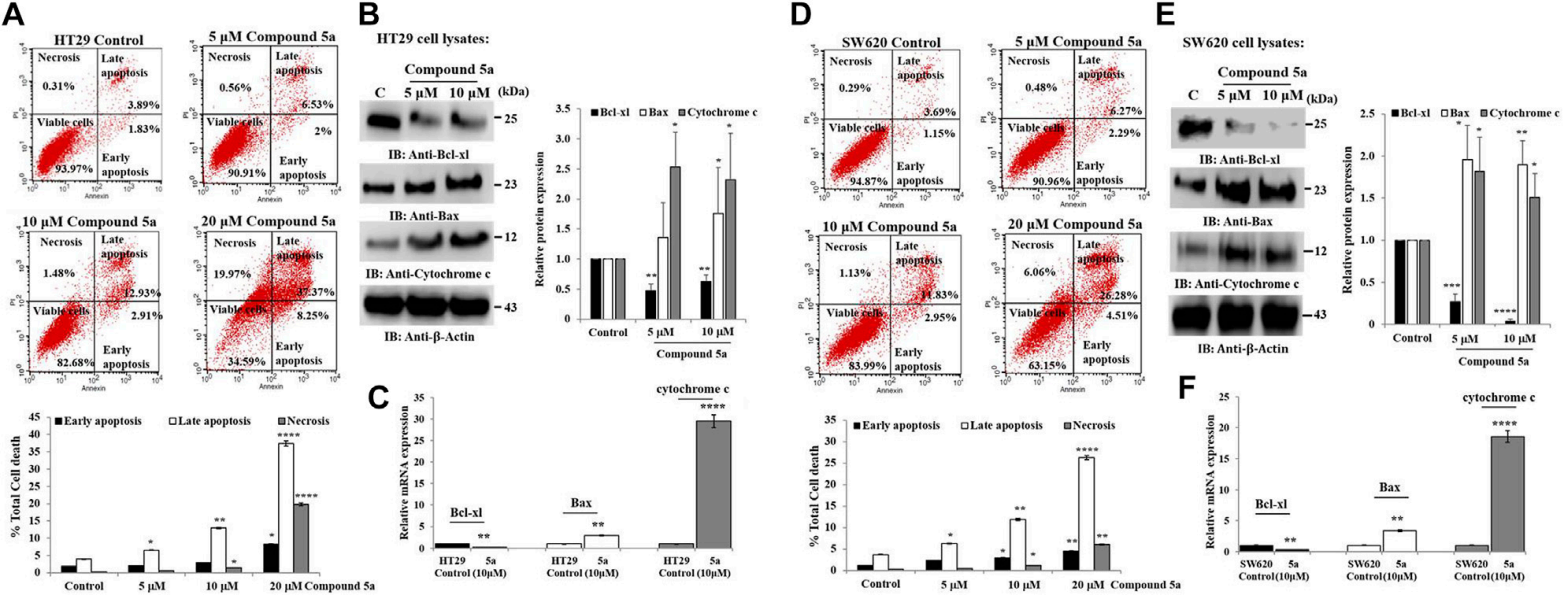
Compounds 5a exerts pro-apoptotic effects associated with Bcl-xl downregulation and Bax and cytochrome c upregulation in HT29 and SW620 cells. Representative scatter plots of apoptotic status determination in HT29 **(A)** and SW620 **(D)** using flow cytometry after Annexin V-FITC/PI-PE double staining. Early and late apoptotic cells are Annexin V+/PI- and Annexin V+/PI+, respectively. Necrotic cells are Annexin+/PI+ and viable cells, Annexin-/PI-. The bar graph depicts the percentage of total cell death, including early, late apoptotic, and necrotic cells. Data are shown as mean ± SD (*n* = 3). Total HT29 **(B)** and SW620 **(E)** cell lysates were immunoblotted against Bcl-xl, Bax, and cytochrome c using their specific monoclonal antibodies. Bar graph indicating the relative Bcl-xl, Bax, and cytochrome c protein expression level, related to the loading control *ß*-actin. Bar graph showing the relative mRNA expression level of Bcl-xl, Bax, and cytochrome c in HT29 **(C)** and SW620 **(F)** RNA extracts related to the internal control GAPDH. Data are shown as mean ± SD (*n* = 3). *p < 0.05, **p < 0.01, ***p < 0.001, and ****p < 0.0001 *vs*. Control.

Compound 5a was reported for its pro-apoptotic activity in HT29 and SW620 cells when tested at 5 and 10 μM (Eldehna et al., 2021c). Here, the cells were exposed at a higher concentration (20 μM) of Compound 5a for a similar incubation time (24 h), revealing a significantly increased induction of apoptosis by 4.0-fold (*p* = 0.00011, Figure 5A) for HT29 cell population and by 2.5-fold (*p* = 0.0001, Figure 5D) for SW620 cell population, compared to the percentage of apoptotic cells detected after cell exposure to 10 μM of Compound 5a (Figures 5A, D). A negligible percentage of necrotic cells was detected in treated HT29 and SW620 cells exposed to 5–10 μM of Compound 5a (Figures 5A, D). However, at 20 μM of Compound 5a, a drastic increase of necrotic HT29 (Figure 5A) and SW620 (Figure 5D) cell populations reaching 20% (*p* = 0.000091) and 6% (*p* = 0.00945) was observed, respectively, compared to the necrotic cell percentage detected in the untreated condition.

**FIGURE 6:**
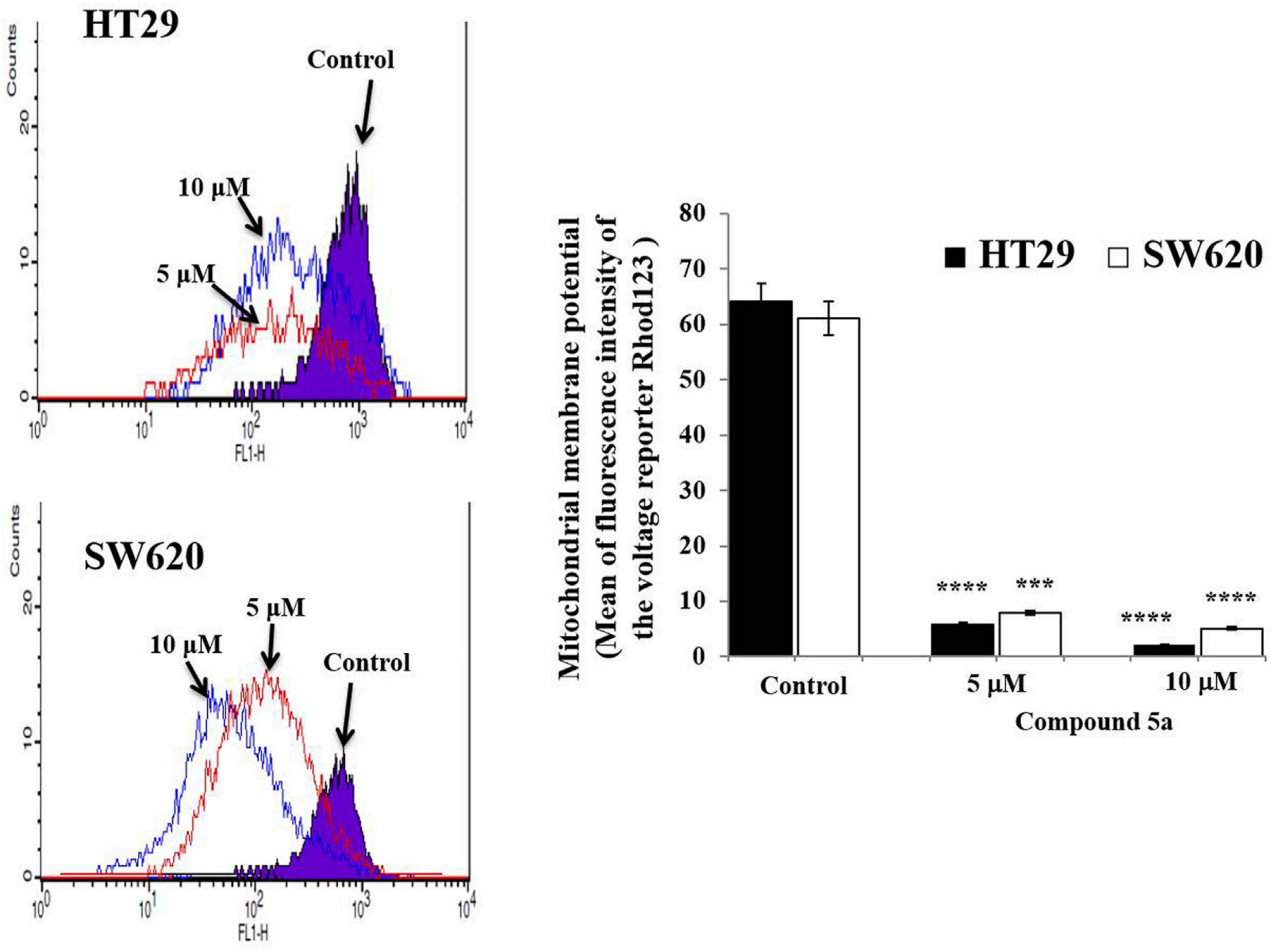
Compound 5a decreases the mitochondrial membrane potential in HT29 and SW620 cells. Representative histograms reveal the mitochondrial membrane potential monitored in untreated and Compound 5a-treated HT-29 and SW620 cells after voltage reporter rhodamine 123 staining and using flow cytometry. The bar graph depicts the mean mitochondrial membrane potential, and the data are presented as mean ± SD (*n* = 3). ***p < 0.001 and ****p < 0.0001 *vs*. Control.

We also previously reported that the observed Compound 5a-induced apoptosis resulted in the suppression of the mitochondrial pro-apoptotic protein Bcl-2 expression and the enhancement of PARP cleavage, suppressing DNA repair (Eldehna et al., 2021c). To study the mitochondrial-dependent apoptosis pathway underlying Compound 5a pro-apoptotic effects more seriously, we first assessed further anti-apoptotic and pro-apoptotic protein expression levels in cell lysates after 5–10 μM of Compound 5a exposure. Playing a key role in the intrinsic apoptotic pathway, mitochondrial membrane potential (MtMP) activity was evaluated using flow cytometry to detect the voltage reporter for mitochondrial membrane potential Rhod123. Regarding the mitochondrial proteins, Compound 5a significantly suppressed by 50% (*p* < 0.01, Figure 5B) Bcl-xl protein expression level in HT29 cells and by about 75% (*p* = 0.00014, Figure 5E) and 90% (*p* = 0.00002, Figure 5E) Bcl-xl expression levels in SW620 cells when tested at 5 and 10 μM, compared to Bcl-xl basal level detected in the untreated cells (Figures 5B,E). A significant upregulation of the pro-apoptotic proteins Bax and cytochrome c expression level was observed in both HT29 (1.75-fold, *p* = 0.015 for Bax; 2.32-fold, *p* = 0.04 for cytochrome c at 10 μM, Figure 5B) and SW620 (1.89-fold, *p* = 0.0069 for Bax; 1.5-fold, *p* = 0.036 for cytochrome c at 10 μM, Figure 5E) cells exposed to 5 and 10 μM of Compound 5a, compared to the basal expression level detected in untreated cells (Figures 5B,E). The downregulation of Bcl-xl and the upregulation of Bax and cytochrome c in HT29 (Figure 5C) and SW620 (Figure 5F) cells exposed to 10 μM of Compound 5a has confirmed at the gene expression levels, compared to basal gene expression levels monitored in untreated cells. Using flow cytometry, the detection of Rhod123, fluorescent dye utilized as voltage reporter for MtMP, was reduced in Compound 5a-treated HT29 and SW620 cells, reflecting a depolarization of the mitochondrial potential, which prompted the release of inner membrane apoptotic proteins (i.e., cytochrome c) to the inter-membrane space then reaching the cytoplasm through permeabilized mitochondrial outer-membrane (Figure 6).

### Compound 5a Enhanced Conventional Chemotherapeutic Drug Cytotoxicity in CRC Cells

**FIGURE 7:**
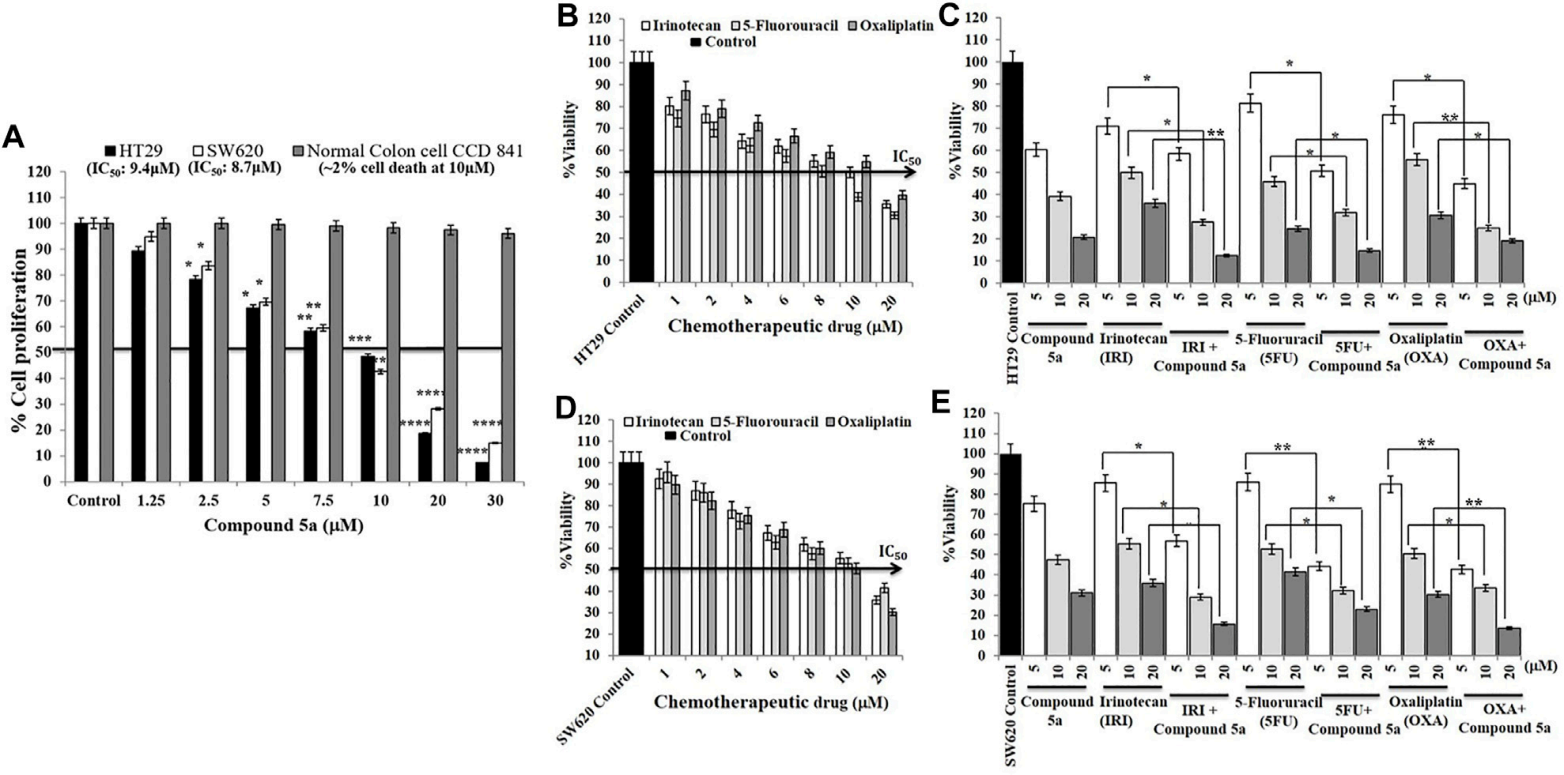
Compound 5a decreases HT29 and SW620 cell viability without affecting the viability of normal colon epithelial CCD 841 cells and enhances irinotecan (IRI), 5-fluorouracil (5-FU), and oxaliplatin (OXA) cytotoxic effects on HT29 and SW620 cells. **(A)** HT29, SW620, and the normal colon epithelial CCD 841 cell lines were exposed to different concentrations (5-10-20 μM) of Compound 5a for 24 h. Cell viability was measured by the MTT assay at 540 nm regarding the cellular metabolic activity. Bar graph showing the cell viability percentage and the data are expressed as mean ± SD (*n* = 3). ***p < 0.001 and ****p < 0.0001 *vs*. Control. Half-maximal inhibitory concentrations (IC50) of Compound 5a on HT29 and SW620 cell viability were determined. HT29 **(B,C)** and SW620 **(D,E)** cells were treated with different concentrations of the chemotherapeutic drugs IRI, 5-FU, and OXA for 24 h in the presence **(C,E)** or absence **(B,D)** of various concentrations (5-10-20 μM) of Compound 5a. Cell cytotoxicity was measured by the MTT assay at 540 nm. Bar graph showing the cell viability percentage and the data are expressed as mean ± SD (*n* = 3). Half-maximal inhibitory concentrations (IC50) of each chemotherapeutic drug on HT29 and SW620 cell viability were also determined.

The 3-(4, 5-dimethylthiazol-2-yl)-2, 5-diphenyl-2H-tetrazolium bromide (MTT) assay has the golden standard for the determination of cytotoxicity caused by any toxic agents on living cells. Adenocarcinoma HT29, metastatic SW620, and normal colon epithelial (CCD841 CoTr) cells were exposed to a wide range of concentrations (1.25–30 μM) of Compound 5a for 24 h, then were subjected to MTT assay. As previously reported, the addition of Compound 5a significantly decreased HT29 and SW620 cell growth, in a dose-dependent manner, compared to the untreated cell growth (Figure 7A). The half-maximal inhibitory concentration (IC50) values were determined as 9.4 μM for HT29 cells and 8.7 μM for SW620 cells (Figure 7A). At all the concentrations tested, the addition of Compound 5a did not affect the growth of the normal colon epithelial cells (Figure 7A). Two percent of cell death was determined when Compound 5a was tested at 10 µM (Figure 7A). In addition to the cytotoxicity caused by Compound 5a, we sought any potentially enhanced cytotoxicity in response to standard drugs, including irinotecan (IRI), 5-fluorouracil (5-FU), oxaliplatin (OXA) combined with Compound 5a. The addition of IRI, 5-FU, and OXA, tested at different concentrations (1–20 μM) and as a single treatment, inhibited the HT29 and SW620 cell proliferation in a dose-dependent manner. IC50 values were determined for each drug on the proliferation of HT29 (10 μM for IRI, 8 μM for 5-FU, 10.7 μM for OXA, Figure 7B) and SW620 (10.4 μM for Iri, 10.1 μM for 5-FU, 10 μM for OXA, Figure 7D) cells, presenting Compound 5a as cytotoxic as these standard drugs. Equimolar chemotherapeutic drugs were combined at a 1:1 M ratio with increasing concentrations (5, 10, 20 μM) of Compound 5a. The combined treatment of IRI with Compound 5a significantly and efficiently inhibited HT29 cell proliferation when tested at 10 µM (−75% *vs*. −50%, *p* = 0.035) and 20 µM (−90% *vs*. −65%, *p* = 0.00924), compared to IRI single treatment (Figure 7C). The combined treatment of 5-FU with Compound 5a significantly and efficiently inhibited HT29 cell proliferation when tested at 5 μM (−50 *vs*. 20%, *p* = 0.042) and 10 µM (−67% *vs*. −55%, *p* = 0.035), compared to 5-FU single treatment (Figure 7C). The combined treatment of OXA with Compound 5a significantly and efficiently inhibited HT29 cell proliferation when tested at 5 μM (−55% *vs*. −25%, *p* = 0.038) and 10 µM (−75% *vs*. −45%, *p* = 0.00945), compared to OXA single treatment (Figure 7C). Concerning mCRC SW620 cells, the combined treatment of IRI with Compound 5a significantly and efficiently inhibited SW620 cell proliferation when tested at 5 μM (−45% *vs*. −15%, *p* = 0.039), 10 µM (−75% *vs*. −45%, *p* = 0.031) and 20 µM (−85% *vs*. −65%, *p* = 0.00975), compared to IRI single treatment (Figure 7E). The combined treatment of 5-FU with Compound 5a significantly inhibited SW620 cell proliferation when tested at 5 μM (−55% *vs*. −15%, *p* = 0.00915), 10 µM (−70% *vs*. −50%, *p* = 0.041) and 20 µM (−80% *vs*. −60%, *p* = 0.039), compared to 5-FU single treatment (Figure 7E). The combined treatment of OXA with Compound 5a significantly and efficiently inhibited SW620 cell proliferation when tested at 5 μM (−55% *vs*. −15%, *p* = 0.00872), 10 µM (−65% *vs*. −50%, *p* = 0.034) and 20 μM (−90% *vs*. −75%, *p* = 0.00934), compared to OXA single treatment (Figure 7E).

## Discussion

Systemic anticancer treatment, such as cytotoxic chemotherapy, requires the urgent development of safe novel drugs, enhancing the efficacy of conventional chemotherapeutic drugs and causing more minor side effects (Redondo-Blanco et al., 2017). Cancer mainly develops through various deregulated processes, including uncontrolled regulation of the cell cycle progression, apoptosis evasion due to the expression of anti-apoptotic proteins overcoming pro-apoptotic protein expression, and dysregulation of DNA repair mechanism due to altered tumor suppressor gene p53 (Feitelson et al., 2015; Marei et al., 2021). At an advanced stage of the solid tumor, such as colorectal carcinoma, the cancer cells develop migratory and invasive potentials, which contribute to metastasis formation and spread the malignant tumor throughout the body to form secondary tumors (Augestad et al., 2015). This invasive stage of the tumor essentially concerns metastatic cells. The search for new anticancer drugs requires potent inhibitory effects on cancer cell migration, invasion, and tumorigenesis. Thus, in this present study, after confirming the reported cytotoxic effect of the synthetic Compound 5a, a benzofuran-isatin conjugate, on colorectal adenocarcinoma HT29 and metastatic CRC SW620 cells (Eldehna et al., 2021c), we showed that Compound 5a exerted anticancer activities by inhibiting CRC cell proliferation, migration, invasion, and colony formation. Compound 5a also reversed epithelial-mesenchymal transition (EMT) phenotype markers by elevating E-cadherin expression and suppressing N-cadherin expression. Furthermore, exposed to Compound 5a, the cells underwent cell death through cell cycle arrest and apoptosis induction, including activation of the intrinsic death-signaling pathway. The combination studies of standard chemotherapeutic drugs irinotecan, 5-fluorouracil, and oxaliplatin with Compound 5a enhanced chemotherapeutic drug efficiency in killing CRC cells, suggesting that Compound 5a is a promising and safe potential for an anticancer chemotherapeutic agent for CRC patients.

Using real-time cell-based assays monitoring the main cell events involved in cancer development and progression, such as cell proliferation, migration, and invasion, the treatment of CRC cells with increasing concentrations (5-10-20 μM) of Compound 5a were monitored at different incubation periods. In most of the cell-based assays, the addition of Compound 5a decreased colon adenocarcinoma HT29 cell proliferation, migration, and invasion, in a dose-dependent manner. Compared to HT29 cells, Compound 5a drastically inhibited all the metastatic CRC SW620 cell functions. The more substantial anti-proliferative effect of Compound 5a in the SW620 cells than its effect in HT29 cells was consistent with a previous study reporting the different levels of CRC cell chemosensitivity to GW4064, a synthetic farnesoid X receptor agonist (Guo et al., 2021). The cancer cell response to the chemotherapeutic drugs is mainly influenced by metabolic alterations in cancer cells caused by oncogenes and tumor suppressors such as p53 (Zaal and Berkers, 2018). In this present study, the expression levels of p53 induced by Compound 5a were higher in the metastatic SW620 cells than in primary adenocarcinoma HT29, which could confirm the more potent anticancer activity of Compound 5a in SW620 cells than in HT29 cells. In addition, differential antitumorigenic effects of Compound 5a were also observed, presenting a drastic decrease of colony formation by SW620 cells than by HT29 cells upon Compound 5a treatment. The EMT phenotype, mainly characterized by E- and N-cadherin expression, plays a crucial role in cancer cell plasticity, tumor progression, and tumorigenicity, as revealed by colony formation (Amawi et al., 2018; Bakir et al., 2020). Herein, Compound 5a upregulated E-cadherin expression while suppressing N-cadherin expression in both CRC cells, confirming the anticancer potential of Compound 5a by restoring E-cadherin expression and downregulating N-cadherin expression as reported by numerous studies (Ma et al., 2016; Pan et al., 2021). However, E-cadherin suppression has also been reported to enhance CRC chemosensitivity to irinotecan and oxaliplatin in HT29 and SW620 cell lines, revealing the complexity of E-cadherin's role in cancer therapy (Skarkova et al., 2021).

Regarding cancer cell migration and invasion, these two processes depending on cell-cell and cell-extracellular matrix adhesion, require the contractility of the cytoskeletal actomyosin, regulated by Rho-associated protein kinase (ROCK) and myotonic dystrophy kinase-related Cdc42-binding kinase (MRCK) activation, and the production and secretion of matrix metalloproteinases, enzymes degrading the extracellular and basement membrane proteins (Lawson and Ridley, 2018). Novel selective multikinase inhibitors of ROCK and MRCK have been reported to efficiently block migration and invasion of various metastatic cancer cells, including melanoma, pancreatic, breast, and colorectal cancer cell lines (Kale et al., 2014; Gandalovičová et al., 2017; Poisson et al., 2020). To confirm the antimetastatic potential of Compound 5a on CRC cells, an assessment of ROCK and MRCK activity would be of great interest.

Cell cycle arrest and apoptosis are the primary targets for anticancer therapy (Sherr and Bartek, 2017; Pfeffer and Singh, 2018). The cell cycle has several well-identified phases (G0/G1, S, and G2/M) through which the cells grow, divide and expand by forming daughter cells (Sherr and Bartek, 2017). In average cell growth, the cell cycle is strictly controlled and mainly regulated by cyclins, cyclin-dependent kinases, and cyclin-dependent kinase inhibitors (Sherr and Bartek, 2017). The G0/G1 phase and the G2/M phase are partly regulated by Cyclin D1 and Cyclin B1, respectively (Tsukahara et al., 2015; Lee et al., 2017). The cyclin A1 is generated in the G1/S phase, contributes to G1/S cell cycle progression, and is rapidly degraded in the G2/M phase (Ji et al., 2005; Thoma et al., 2021). G2/M phase arrest is an important cell cycle checkpoint to stop the cells harboring damaged DNA from entering mitosis (M phase), which triggers DNA repair or apoptosis upon p53 activation (Jeong et al., 2015). Thus, G2/M phase arrest is a frequent consequence of anticancer drugs interfering with DNA replication (Ramadan et al., 2020). Anticancer compounds can cause S phase arrest by interfering with the Cyclin A1/Cyclin-dependent kinase (Cdk)2 complex (Bai et al., 2017). Cell cycle arrest in tumor cells is regularly accompanied by cell proliferation inhibition (Otto and Sicinski, 2017). Flow cytometry was carried out to study the effect of Compound 5a on the cell cycle phase distribution after a 24-h CRC cell exposure. Compound 5a prompted G1/G0 phase arrest in HT29 cells and G2/M phase arrest in SW620 cells, while a decrease in HT29 and SW620 cell population in the S phase was observed. Upon Compound 5a treatment, the downregulation of cyclin A1 in both CRC cells confirmed the interference of the entry in the S phase. However, a p53-induced gene (Rivera et al., 2006), an upregulation of cyclin A1 expression was expected, suggesting further transcriptional studies. In addition, the Compound 5a-induced Cyclin D1 and B1 downregulation at similar protein expression levels in both CRC cells could not explain the difference in cell cycle arrest, requiring further investigation of the critical cell cycle regulators of the G0/G1 phase and G2/M phase. A complex process occurred in activating the extrinsic death receptor-dependent and intrinsic signaling pathways, and flow cytometry was applied for apoptosis determination and MtMP evaluation. Herein, after confirming the reported pro-apoptotic effect of Compound 5a, an increase in apoptotic cells was observed when both CRC cells were exposed to a higher concentration (20 μM) of Compound 5a (Eldehna et al., 2021c). At lower concentrations (5–10 μM), Compound 5a doubled (i.e., 10 *vs*. 5 μM) the percentage of apoptotic CRC cells, which aligned with Compound 5a-induced p53 upregulation, tumor suppressor protein well described to mediate apoptosis (Marei et al., 2021). The mitochondrial cytochrome c, the pro-apoptotic (Bax), and anti-apoptotic proteins (Bcl2, Bcl-xl) are critical determinants of the cell fate towards cell survival or cell death under stress (Kalkavan and Green, 2018). In this study, Compound 5a induced the expression of the cytochrome c and pro-apoptotic protein Bax while downregulated Bcl-xl in human adenocarcinoma HT29 and metastatic CRC SW620 cells. Thus, the induction of Bax expression and the inhibition of Bcl-xl expression provide a mechanistic basis for Compound 5a-induced apoptosis. Mitochondria are considered a prime location where cellular stress signals converge, leading to the execution of apoptosis (Galluzzi et al., 2012). Loss of the mitochondrial membrane potential is an early event in the intrinsic apoptotic pathway (Pfeffer and Singh, 2018). Compound 5a treatment resulted in a significant loss of mitochondrial membrane potential in both CRC cells, leading to the release of cytochrome c into the cytosol culminating in caspase activation. The detection of cleaved effectors caspase-3/7, caspase-9, and caspase-8 in Compound 5a-treated CRC HT29 and SW620 cells would verify and validate the apoptotic status, the intrinsic and death-receptor-dependent pathway activation, and respectively.

The anticancer efficacies of current therapeutics are limited because of the high degree of cancer clonal heterogeneity and intratumor genetic variation, especially in colorectal cancer (Ramón y Cajal et al., 2020). Therefore, using combinations of molecular-targeted agents has increased for better therapeutics. Drug-drug interaction can pose serious patient side effects (Moghaddas et al., 2021). Thus, drug treatment, dosages, and combinations given are often decided empirically based on evidence of *in vitro* studies and reported clinical outcomes (Cheng et al., 2019). Thus, the knowledge of the initial functional responses of cells to drugs remains helpful in determining the most efficacious use of drugs or their combinations to treat cancer. The effectiveness of the anticancer drugs and the possible interactions of these drugs in combined therapies for cancer cell proliferation inhibition are requested, including drug antagonism and synergism. The exchange could be pharmacokinetic (PK) and pharmacodynamics (PD). The PK interactions result in altered drug distribution, absorption, and elimination. Pharmacodynamics interactions cause alterations in the way a drug or compound affects a tissue or organ system. In this study, lower doses of individual drugs could be used in combination than when given separately and at specific combined doses resulting in synergism.

## Conclusion

Our findings described the promising *in vitro* anticancer activities of the synthetic benzofuran-isatin conjugate Compound 5a in colorectal adenocarcinoma and metastatic CRC cell lines resulting in inhibition of the primary cancer cell events involved in tumor development (i.e., cell proliferation, colony formation, cell cycle) and progression (i.e., migration, invasion, reversed EMT phenotype markers). Its pro-apoptotic effects are demonstrated by the downregulation of the anti-apoptotic protein Bcl-xl, upregulation of the pro-apoptotic proteins Bax and cytochrome c, and mitochondrial outer membrane permeabilization, which suggest that Compound 5a is a safe anticancer drug. The studies of a potential combination treatment of Compound 5a with conventional chemotherapeutic drugs enhanced their efficiency in eliminating CRC cells, suggesting Compound 5a as a promising and safe potential anticancer chemotherapeutic agent for CRC patients. However, *in vivo* studies are still needed.

## Data Availability

The raw data supporting the conclusion of this article will be made available by the authors without undue reservation.
